# Drug safety monitoring in patients of movement disorders of a tertiary care hospital

**DOI:** 10.4103/0253-7613.68437

**Published:** 2010-08

**Authors:** Ananya Mandal, Suparna Chatterjee, Shyamal Kumar Das, Amar Mishra

**Affiliations:** Department of Pharmacology, Nilratan Sircar Medical College & Hospital, Kolkata - 700014, India; 1 Department of Pharmacology, Institute of Postgraduate Medical Education & Research, Kolkata - 700 020, India; 2 Department of Neuromedicine, Bangur Institute of Neurosciences & Psychiatry, Kolkata-700025, India

**Keywords:** Adverse drug reactions, drug-induced movement disorders, movement disorders, pharmacovigilance

## Abstract

**Background:**

Movement disorders (MD) are neurological conditions that affect the speed, fluency, quality, and ease of movement and commonly include Parkinson’s disease, tremor and dystonias. Drugs are important causes of MD, and the incidence and prevalence of such disorders are possibly underappreciated because of the lack of recognition.

**Objectives:**

To assess the incidence of all adverse drug reactions (ADRs) and estimate the prevalence of drug-induced MD among patients attending the clinic.

**Materials and Methods:**

This prospective observational study was conducted at an outpatient referral MD clinic of a tertiary care hospital for 1 year. The demographic data, drug intake, diagnosis, and ADRs experienced by the subjects were recorded. Causality assessment was done by Naranjo’s scale.

**Results:**

Incidence of ADR among patients who attended this clinic was 19.7% (151 out of 768 patients experienced at least one ADR). A total of 299 ADRs were detected out of which 30.8% were gastrointestinal, 28.4% psychiatric, and 26% MD effects. The commonly implicated suspect drugs were levodopa (37.8%) and trihexyphenidyl (25.1%). The prevalence of drug-induced MD was 10.15% and drug-induced dyskinesias and dystonias were the most common.

**Conclusion:**

MDs are clinically important neurological disorders which are often caused by drugs and interestingly drugs used for its management are also associated with high incidence of ADRs. Hence these ADRs should be carefully monitored.

## Introduction

Movement disorder (MD) constitute the neurological conditions that affect the speed, fluency, quality, and ease of movement and commonly include Parkinson’s disease, Wilson’s disease, tics, Gilles de la Tourette’s syndrome, myoclonus, essential tremors, dystonias, ataxia, and drug-induced MDs.

Incidence of Parkinson’s disease in India varies from 68 to 328.3 cases per 100,000 population;[1] dystonias (0.17 to 5 per 100,000); tics (2 per 100,000 population).[2] Drug-induced MD contribute significantly to such ailments.[3]

Since these disorders generally require therapy with multiple drugs for prolonged periods, drug-related adverse effects and other coexisting ailments often add on to the existing morbidity.[3] Although, such adverse drug reactions (ADRs) are common, comprehensive information about their incidence, severity, and ultimate health effects is not available. A MEDLINE search reveals that till date there are no reported studies assessing the safety of the drugs commonly used in such clinical setting especially in India. However, there are published pharmacoepidemiological studies from other countries on drug usage patterns in Parkinson’s disease.[4,5] We therefore undertook this study to assess the incidence of all ADRs and the prevalence of drug-induced MD ADRs in patients attending this specialized clinic.

## Materials and Methods

This was a prospective, observational study conducted at the outpatient clinic of a tertiary care neurosciences teaching hospital in Kolkata. All patients who attended this weekly clinic during this 1 year period were monitored. All suspected spontaneous ADRs were initially assessed by the neurology consultants and subsequently the information was analyzed by pharmacologists. Detailed clinical and drug history and relevant information about the suspected reaction, its onset, duration, temporal association with drug intake if any, concomitant drug therapy, past history, reports of relevant laboratory investigations undertaken to arrive at the clinical diagnosis were recorded in an ADR reporting form (a modified version of the National Pharmacovigilance Program ADR reporting form). The reactions were later categorized and causality assessment was done using the Naranjo’s ADR probability scale.[6] Data were compiled on a Microsoft Access Database and analyzed statistically.

## Results

768 patients attended the clinic during the study period (November 2006--October 2007) and 151 patients experienced at least one ADR (incidence rate of 19.7%). A total of 299 ADRs were recorded (mean 1.98 ADR/case). The mean age of patients who experienced ADRs was 47.34 years with a male preponderance (68.6%). The system-wise categorization of the ADRs detected and drugs implicated are enlisted in Table 1.

**Table 1:** Categories of adverse drug reactions detected and implicated drugs

*Type of ADRs (number of ADRs)*	*Drugs implicated (in order of frequency)*
Gastrointestinal (92)	Trihexyphenidyl, levodopa, escitalopram, amantadine, phenytoin, penicillamine, pramipexole, topiramate, risperidone, entacapone
Psychiatric (85)	Levodopa, trihexyphenidyl, tetrabenazine, clonazepam, escitalopram, clozapine, selegiline, pramipexole, ropinirole, propranolol, haloperidol, topiramate, zolpidem, olanzapine, clonidine
Movement disorders (78)	Levodopa, haloperidol, trihexyphenidyl, phenytoin, risperidone, quetiapine, olanzapine, ropinirole, clonazepam, chlorpromazine, bromocriptine, donepezil, metoclopramide, entacapone, pramipexole, clozapine, valproate, tetrabenazine
Cardiovascular (13)	Levodopa, amantadine, propranolol, haloperidol, escitalopram, penicillamine
Neurological (9)	Trihexyphenidyl, amantadine, phenytoin, selegiline, tetrabenazine
Genitourinary (5)	Trihexyphenidyl, levodopa, risperidone
Cutaneous (2)	Levodopa, haloperidol
Ocular (2)	Trihexyphenidyl
Others (13)	Amantadine, sertraline, trihexyphenidyl, tetrabenazine, olanzapine, valproate, propranolol, clozapine

Gastrointestinal ADRs were the most common, accounting for 30.8% of the total reactions and included anorexia, constipation, nausea and vomiting, hypersalivation, dry mouth, and dysphagia. These ADRs mainly required dose titration or withdrawal of the suspect drug. Neuro-psychiatric ADRs were the next common type (28.4%) and included *hypersomnolence*, insomnia, paranoid ideation, hallucinations, psychosis. Dopaminergic precursor levodopa and agonists---ropinirole, pramipexole, MAOB inhibitor---selegiline, anticholinergic---trihexyphenidyl, and antipsychotics (both typical and atypical) were the main suspect medications. Other neurological ADRs included memory impairment and cognitive dysfunction associated with long-term levodopa therapy (on therapy >2 years). Although these are relatively rare ADRs associated with levodopa, some studies have however reported them.[7–9]

MD ADRs accounted for 26.08% of the total and included dyskinesias, dystonia, tremors, ataxia, akathisia, akinesia, tics, hemiballismus, and myoclonic jerks.

ADRs affecting other organs and systems were lesser in frequency. The most common cardiovascular ADR encountered was levodopa-induced postural hypotension. Pedal edema and levedo reticularis with amantadine were also seen. Genitourinary ADRs like urinary retention and ocular effects like blurred vision and dryness of eyes were encountered with trihexyphenidyl. ADRs with rare incidences in our study included cases of agranulocytosis, weight gain, dyspnea, urticarial rash, raised liver enzymes, and dyspnea.

Analysis of the data showed that the top five common offending drugs causing ADRs were levodopa (37.8%) and trihexyphenidyl (25.08%), escitalopram (5%), amantadine (4.3%), and haloperidol (3.3%).

48 serious ADRs were detected and were categorized as those leading to significant physical disability requiring withdrawal of the suspect drug, active treatment, and institution of alternative drugs. However no deaths or hospitalization due to the ADRs were required. The incidence of serious ADRs appear to be high as most of the cases were difficult to treat and complicated ones requiring multiple drug therapy.

The incidence of drug-induced MD was (78/786) 10.15%. The different categories of drug-induced MD detected in the clinic were dyskinesias, dystonias, ataxia, tremors, and tics. A majority of the drug-induced disorders were chronic or late onset ones like tardive dyskinesias, Parkinsonism, and dystonias, while acute onset disorders like extrapyramidal symptoms were very few and no cases of malignant neuroleptic cases were detected. Antipsychotic drugs were the most commonly implicated drugs causing MD and included both typical antipsychotics---haloperidol, risperidone and atypical ones olanzapine, quetiapine, and clozapine. Dopaminergic agents like levodopa were also an important cause of various MDs. Average number of drugs prescribed/patient was 3.7 and the average duration of suspect drug use at diagnosis was 36 months.

## Discussion

This prospective observational study was undertaken with the aim of assessing the incidence of all ADRs and drug-induced MD in this specialized MD clinic for 1 year. The incidence of ADRs was found to be 19.7% in the population encountered. In the absence of any published Indian study in this area we compared the results of our study with one which reported 14% of all detected ADRs were neurological.[10,11]

The incidence of ADRs in our study appears to be high due to the study setting which was a referral, specialized clinic that catered mainly to relatively non-responsive or difficult to treat complicated cases. Most of the patients were on multiple drugs which could have increased the likelihood of development of ADRs. Most of the ADRs reported in this study however were known.

The incidence of drug-induced MD in this study was 10.15% indicating that drugs are an important cause of serious MD and the results of this study are comparable with some previously published studies. A study analyzed the incidence of only antipsychotic induced MDs and showed that dystonias, tardive dyskinesias, parkinsonism, akathisia were the commonly detected ones while neuroleptic malignant syndrome were rare.[12] In our study the commonest type of drug induced MD detected was dyskinesia, dystonia, Parkinsonism and tremors. We however, did not encounter any case of malignant neuroleptic syndrome. The true incidence and prevalence of drug-induced MDs is very difficult to estimate because of lack of recognition. The diagnosis of drug-induced MD is often a difficult one, as the clinical features are the same as that for the primary MD of other etiology. The diagnosis was based on the recognition of the abnormal movement, subsequently establishing the temporal relationship between drug therapy, and improvement with dechallenge in selected cases or substitution therapy.

One of the major limitations of this study was that firstly, the patients attending this special clinic may not allow us to truly judge the background incidences of these ADRs. Secondly, the causality assessment which was done by Naranjo’s Scale revealed that most cases were of “probable” or “possible” association and no cases with “definite” association were detected.

MDs are a spectrum of clinically important neurological disorders which are often caused by drugs, and interestingly drugs used for its management are also associated with high incidence rates of ADRs. The high incidence of ADRs could be attributed to either polypharmacy or the chronic nature of the underlying disorders requiring long-term drug usage. This study therefore suggests that drugs are an important etiological factor for MD and clinicians should make an attempt for early case detection and be vigilant about safety profile monitoring of the prescribed medications.
